# Biomarkers for Detection and Monitoring of B16 Melanoma in Mouse Urine and Feces

**DOI:** 10.1155/2015/841245

**Published:** 2015-02-23

**Authors:** Aviv Sever, Amir Abd elkadir, Yosef Matana, Jacob Gopas, Yehuda Zeiri

**Affiliations:** ^1^Department of Biomedical Engineering, Ben-Gurion University of the Negev, 84105 Beer-Sheva, Israel; ^2^The Shraga Segal Department of Microbiology, Immunology and Genetics, Ben-Gurion University of the Negev, and the Department of Oncology, Soroka University Medical Center, 84105 Beer-Sheva, Israel; ^3^Division of Chemistry, Nuclear Research Center-Negev, 84190 Beer-Sheva, Israel

## Abstract

Melanoma is the most malignant type of skin cancer. Early detection of melanoma is thus critical for patient prognosis and survival. At present, examination by a skilled dermatologist followed by biopsy of suspicious lesions is the diagnostic gold standard. The aim of the present study was to examine an alternative and noninvasive method for the diagnosis of melanoma at an early stage. We identified and compared the volatile organic compounds (VOCs) in mouse urine and feces, before and after a subcutaneous injection of B16 melanoma cells. We identified a total of 16 VOCs in urine and 13 VOCs in feces that could serve as potential biomarkers. Statistical analysis significantly discriminated between the cancer and control groups. These results should be validated in a larger-scale animal study, after which a study could be designed in patients to develop a melanoma biomarker.

## 1. Introduction

Early detection of malignant melanoma is critical for lowering the mortality of this cancer. We explore, in mice, the possibility of diagnosing an early-melanoma tumor and following its growth by analyzing the changes that occur throughout the pathological process in VOCs excreted in urine and feces. We report here total of 29 potential biomarker candidates of melanoma; 25 of these are novel, and 4 have been previously reported by other research groups in relation to VOCs detected from cultures of human melanoma cells. The chemical identity of only 24 of the 29 possible biomarker candidates was revealed with high confidence. This study will contribute to the development of a noninvasive and reliable diagnostic procedure to detect melanoma in the urine of patients.

Melanoma accounts for less than 2% of all skin cancers, but for the vast majority of deaths from skin cancer. In the United States alone, it is estimated that 76,100 individuals will be diagnosed with melanoma during 2014 and that 9,710 patients will die from it [1]. Early detection of malignant melanoma is the key factor in reducing mortality from this cancer. Although histopathological examination is the gold standard for diagnosis of melanoma, additional, noninvasive approaches should be developed [2]. Here, we explore the possibility of exploiting volatile organic compounds (VOCs) to develop such an approach; hundreds of VOCs are emitted from the human body and usually reflect the metabolic condition of the individual. Therefore, pathological processes, such as cancer, are expected to influence the VOC fingerprint of patients either by changing the ratio between different VOCs or by producing new VOCs [3].

It has been reported that dog scan identifies, by using their olfaction sense, melanoma on the skin of patients [4] or melanoma samples hidden on healthy subjects [5]. These findings suggest that VOCs from melanoma differ from those of normal skin. D'amico et al. [6] used gas sensor array (also known as an “electronic nose”) to differentiate between melanomas and nevus lesions. Those authors compared the lesion with the adjacent skin region to reduce the skin headspace variability. The array discriminated between the different lesions with an accuracy of about 80%. Abaffy et al. [7] collected biopsy samples and compared the VOCs released from melanoma tissue to those released from nevus and normal tissue, using matching skin as a control. Those authors found 32 potential biomarker candidates; the concentration of 9 VOCs increased in the presence of melanoma, and 23 VOCs were detected only when melanoma cells existed and were not detected in normal cells. The authors used the NIST 2.0 mass spectral database, with a 60% quality factor for chemical identification of the possible biomarker candidates; however, they did not report the quality factor obtained for the different compounds by mass spectrometry (MS). In a recent study, Kwak et al. [8] compared human melanoma cells to normal melanocytes cultured in vitro. By using gas chromatography- (GC-) MS, those authors found increased levels of both isoamyl alcohol and isovaleric acid in the headspace over melanoma cells. The authors also found that the melanoma cells produced some unique compounds, such as dimethyldisulfide and dimethyltrisulfide.

In the present study, we analyzed the VOCs emitted from the urine and feces of mice before (healthy group) and after (cancer group) subcutaneous injection of B16 melanoma cells. We also analyzed the volatiles emitted by B16 melanoma cells culture. The present work will serve as a basis to analyze possible VOCs in human patients. We expect some VOC to be shared between the species and new and different VOCs to be detected.

## 2. Results

### 2.1. Comparative Analysis of the VOCs Obtained from Healthy and Tumor-Bearing Mice

**Figure 1:**
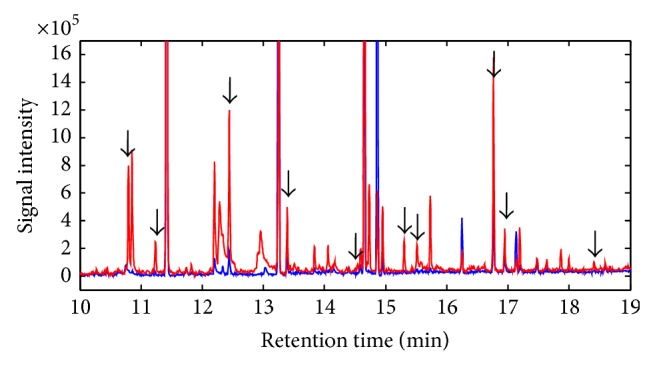
Chromatograms obtained from urine sample of one typical mouse before (blue line) and after (red line) the injection of B16 melanoma cells. Each peak in the chromatogram represents a compound, and the peak area is proportional to the concentration of the VOC. The arrows indicate differences that were found to be significant.

The sample classification used in the present study is summarized in Table 1. The analysis of the urine samples of healthy and tumor-bearing mice revealed some differences in the VOCs composition. There was no significant difference between the first two samples (samples A and B), which were taken before the injection of the B16 melanoma cells, and the first sample taken after injection (sample C). In the second sample taken after the injection (sample D), a significant difference was found in the observed VOCs, although tumors were not yet palpable. In the last two samples (samples E and F), tumors were palpable in all mice, and the chromatographic spectra were significantly different from those obtained in earlier samples. Therefore, the samples were classified into three groups: healthy, early-melanoma, and late melanoma. Typical urine chromatograms obtained from one mouse before (sample B) and after injection (sample E) are shown in Figure 1. Some peaks in the chromatograms were present only in the tumor-bearing mice, and the area under some peaks was markedly higher or lower in the tumor-bearing mice compared to the healthy mice.

By using the NIST'08 and Wiley mass spectral libraries, total of 120 VOCs were identified in urine and 139 VOCs in feces were identified in all mice samples, with an 80% quality factor (QF). After subtracting the VOCs that were found in the background samples, 66 VOCs in urine and 75 VOCs in feces remained. A statistical analysis reduced the numbers of these peaks to 16 VOCs in urine and 13 VOCs in feces, which could serve as possible biomarker candidates. In addition, three VOCs in urine and two VOCs in feces, with a QF in the range of 60–79%, were also included as possible biomarker candidates.

Of the 19 possible biomarker candidates in urine, three compounds were detected only in the tumor-bearing mice (Table 2), three VOCs were detected only in the healthy and early-melanoma-bearing mice (Table 3), and 13 VOCs were detected in markedly higher concentrations in the tumor-bearing mice as compared with the healthy group (Table 4). Similarly, from the 15 possible biomarker candidates found in the headspace of feces, two VOCs were detected only in the tumor-bearing mice (Table 5) and 13 VOCs were found with a markedly higher (12 VOCs) or lower (1 VOC) concentration in the tumor-bearing mice than in the healthy group (Table 6). To examine the significance of the changes in the concentrations of compounds during the different melanoma stages (see Tables 4 and 6), a two-tailed paired *t*-test was carried out.

As can be seen in Table S1 in Supplementary Material available online at http://dx.doi.org/10.1155/2015/841245, some of the VOCs that were identified as possible biomarker candidates in the current study have already been reported previously by other research groups as being predictive for melanoma and other cancers. The four potential melanoma-related biomarkers that have already been reported are isopropyl palmitate (RT = 18.41, QF = 72%), 1-hexadecanol (RT = 14.54, QF = 93%), benzaldehyde (RT = 6.94, QF = 97%), and dimethyl sulfone (RT = 6.21, QF = 94%). 1-Hexadecanol and isopropyl palmitate, a derivate of palmitic acid, were detected only in the urine of tumor-bearing mice. These findings are consistent with a previous study [7], which reported a 35-fold increase in the level of 1-hexadecanol in the case of melanoma (as compared with a matching skin sample) and that isopropyl palmitate was found only in the melanoma sample. The authors suggested that these compounds may reflect an increased* de novo *synthesis of fatty acids, which is a crucial metabolic alteration that cancer cells require for synthesis of a new plasma membrane.* De novo *synthesis of fatty acids could be caused by the hyperactivity of the oncogenic fatty acid synthase (FASN), a common phenotype in cancer pathogenesis [9]. Benzaldehyde levels in feces were 132% higher in the tumor-bearing mice than in the healthy group (*P* = 0.004), but it is important to notice that the increases were from the early-melanoma stage to the late melanoma stage (*P* = 0.002), and not from the healthy group to the early-melanoma stage. In other words, benzaldehyde could be a useful possible biomarker candidate for predicting melanoma, but not in its early stage. The levels of dimethyl sulfone in urine showed a 199% increase from the healthy to the tumor-bearing mice groups (*P* = 0.026). As shown earlier [8], significantly higher amounts of this compound were found in metastatic melanoma cells, as compared with normal cells. This finding suggests that the metabolism of sulfur-containing amino acids by melanoma cells differs from that in normal cells.

### 2.2. Chemical Analysis of VOCs Released from B16 Melanoma Cell Cultures

The GC-MS analysis of headspace in B16 melanoma cell cultures yields 12 VOCs that were not detected in the headspace of the materials in the phosphate buffered saline (PBS) and fresh growth medium without cells (Table 7). All these compounds were identified with QF that was higher than 80%. The compounds that were identified were isopropyl myristate (RT = 16.76, QF = 93%), decane (RT = 7.43, QF = 93%), 2,4-dimethyl-1-heptene (RT = 4.67, QF = 93%), and hexadecane (RT = 14.7, QF = 95%). As described above, the concentrations of isopropyl myristate were higher in both urine and feces of tumor-bearing mice. Decane and two chemically similar compounds, 1-hexadecene and 2,4-dimethyl-heptane, were previously reported [7] to be present only in melanoma samples. The difference between 1-hexadecene and 2,4-dimethyl-heptane to hexadecane and 2,4-dimethyl-1-heptene (resp.) is in the presence or absence of a single double bond. Hakim et al. [10] studied two hydrocarbons, decane and 2,4-dimethyl-1-heptene, that were reported as potential biomarkers of lung cancer and suggested that these compounds are probably the outcome of oxidative stress.

### 2.3. Monitoring Melanoma Stage

**Figure 2:**
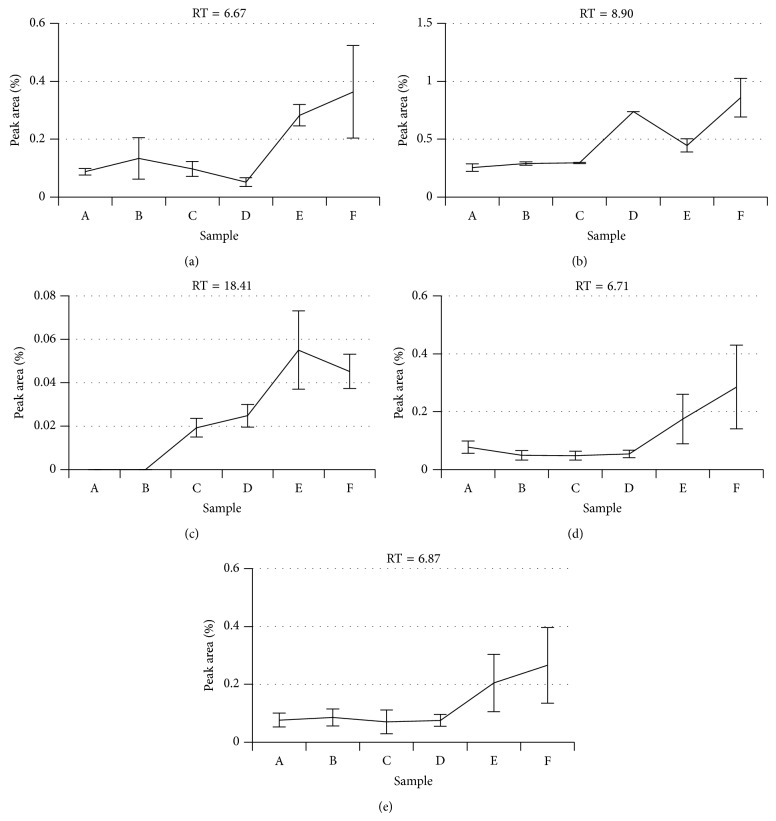
Potential biomarker candidates that exhibited a marked increase in concentration as tumor volume increased. Data are expressed as means ± standard deviation. (a)–(c) Urine potential biomarkers. (d) and (e) Feces potential biomarkers. (Note: the concentration is proportional to percent of peak area relative to the total chromatogram area.)

As mentioned above, the tumor was palpable only in the last two samples (late melanoma). An average lesion volume of 1664 mm^3^ (*n* = 2) was measured three days after the last sample was taken (see Figure S1). Among all possible biomarker candidates that were found in the urine and feces, three compounds in urine (Figures 2(a)–2(c)) and two compounds in feces (Figures 2(d)-2(e)) exhibited a marked increase in concentration as a function of tumor growth. Three of these compounds were all ketones, that is, 5-methyl-2-heptanone (RT = 6.87, QF = 91%), 6-methyl-2-heptanone (RT = 6.71, QF = 94%), and 6-methyl-3-heptanone (RT = 6.67, QF = 97%). The quality factor of the two compounds found in the feces headspace was smaller than 80%; hence they are referred to only by their retention time: RT = 8.9 and RT = 18.41. 6-Methyl-2-heptanone has been found in the blood of patients with liver cancer [11] and 6-methyl-3-heptanone was found in the urine of mice with lung cancer [12]. In addition, other chemically similar ketones, such as 2-heptanone and 6-hydroxy-6-methyl-3-heptanone, were also detected in urine samples from mice with lung cancer tumors [13].

### 2.4. Statistical Models

**Figure 3:**
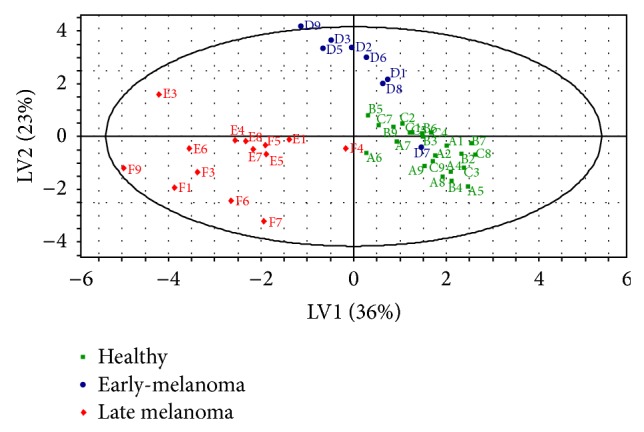
Score plot for the urine PLS-DA classification model with 3 latent variables. Sixty-eight percent of the variance in the data (*R* ^2^ = 0.68) are explained, and 73% of the data are predicted (*Q* ^2^ = 0.73), by this model. A very good discrimination can be observed between the “healthy,” the “early-melanoma,” and the “late melanoma” clusters. Letters represent suggested classification (see Table 1) and numbers indicate mouse identity (1–9).

**Figure 4:**
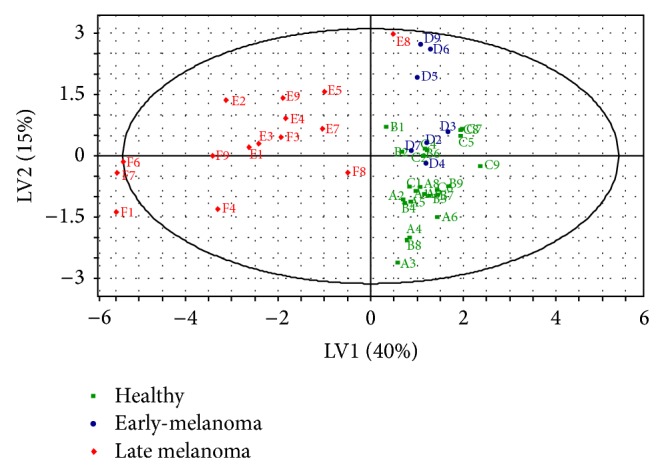
Score plot for the feces PLS-DA classification model with 3 latent variables. Sixty-eight percent of the variance in the data (*R* ^2^ = 0.68) is explained, and 61% of the data are predicted (*Q* ^2^ = 0.61), by this model. A good discrimination can be observed between the “healthy” and “early-melanoma” clusters to the “late melanoma” cluster. Letters represent suggested classification (Table 1) and numbers indicate mouse identity (1–9).

Partial least square discriminate analysis (PLS-DA) models of urine and feces were built with the SIMCA P+ software. Each model included three latent variables that were calculated by the software and are, in fact, weighted linear combinations of the 12 possible biomarker candidates that most contributed to the separation between the three groups (all the compounds in Tables 2–6, except compound 2 in Table 2, compound 3 in Table 3, compounds 3 and 9–12 in Table 4, compound 1 in Table 5, and compounds 5 and 6 in Table 6). The models are shown in Figures 3 and 4 for the urine and feces models, respectively. The models were validated by using 7-fold cross validation [14]. The validation results are shown in Table 8 for the urine and feces samples. The urine model exhibited good predictability of the mouse condition (healthy or sick) with a 92.3% sensitivity, 100% specificity, a 93.1% negative predictive value (NPV), and a 100% positive predictive value (PPV). The model for feces gave less significant results, with an 80.8% sensitivity, a 96.3% specificity, an 83.9% NPV, and a 95.5% PPV. It is important to note that eight out of nine mice were correctly predicted with an early-melanoma by using the urine model, whereas the feces model did not clearly discriminate between “healthy” and “early-melanoma” mice.

## 3. Discussion

The present study constitutes a novel proof-of-concept for the detection and monitoring of melanoma in urine and feces samples of mice at an early stage. Our urine statistical model correctly predicted eight out of the 9 mice bearing an “early-melanoma,” indicating that mice at that stage can be detected and distinguished from healthy mice and from mice bearing a “late melanoma.”

While other research groups [6, 7] focused on comparing human melanoma to normal and nevus skin biopsies, we attempted to distinguish between mice with an early-melanoma and healthy mice. Our model was able to distinguish between melanoma bearing mice and healthy mice with sensitivity of 92% and specificity of 100%, as compared to studies of other groups on human samples (e.g., 70% sensitivity and 90% specificity reported by [6] and 89% sensitivity and 90% specificity reported by [7]). These differences might be related to the low variability in the VOCs of mice all having similar genetics and environment, as compared to those of humans.

The results of this study also indicate a marked difference between healthy mice and mice bearing a “late melanoma,” whereas the difference between healthy mice and mice bearing an “early-melanoma” is less clear. One possible explanation for this finding is that the concentration of the “new” compounds, which are products of the altered metabolism of the melanoma cells or of the normal cells that it affects, is below the detection threshold of our GC-MS system. In addition, the angiogenesis in mice bearing an “early-melanoma” is limited and, therefore, metabolites induced by melanoma cell do not disperse efficiently and are thus hard to detect. To overcome these concentration-dependent limitations, one should concentrate on detecting the compounds (potential biomarker candidates) that are responsible for the differences between healthy and “late melanoma” stages and analyzing urine only as a screening test. One should also develop methodologies to detect VOCs directly from the regions of skin showing suspicious lesions; a step in this direction has been taken by detecting differences in human melanoma cell line signatures by FTIR spectroscopy [15, 16].

Some of the potential biomarker candidates that were identified in the present study were previously reported to be predictive for melanoma and other malignancies (Table S1). Those reports strengthen the validity of our findings and imply the existence of “cancer type”-specific biomarkers and of common cancer biomarkers. Some of the potential biomarker candidates are cross species compounds; namely, they are found in both mice and human melanoma cells; therefore, similar experiments with human melanoma cell lines as xenografts should be conducted to confirm these data. Two additional possible biomarker candidates that may be good candidates to identify melanoma are dehydroabietic acid (RT = 21.7) and 2-hexanone (RT = 3.78). Dehydroabietic acid is a toxic and allergenic compound found in wood resins. It could be a pollutant from the sawdust, although it was not detected in the background samples. Another possibility could be wrong identification of this compound by the code used MS database search (QF = 81%). Importantly, isopropyl myristate, the concentration of which increased in the urine and feces of tumor-bearing mice, was also found in the vapor phase of the B16 melanoma cells culture. This finding suggests that isopropyl myristate is a valid direct product of melanoma cell metabolism for further study. Other compounds that were found in the headspace of the melanoma cell cultures were not detected in the urine or feces samples, and vice versa. This is attributed to the differences between the tumor cells microenvironment in a culture as compared to a tumor in the animal. We assume that there may be also contribution from the tumor microenvironment since tumor cells affect other cells in the tissue. At this point, the origin of the specific molecules we detected cannot be determined. The observed increase in the concentration of ketones as a function of tumor growth suggests that ketogenesis pathways, incorporating various ketone formations, may be involved in the model of melanoma cancer. Therefore, monitoring these ketones could help in tumor follow-up in melanoma patients. Finally, to substantiate the findings in this study, we plan to perform an extended study with larger number of mice focusing on the urine VOCs. In addition, to correlate more precisely between tumor size and VOC concentration one should collect samples more often (every 2–4 days) and correlate the biomarkers to tumor volume as measured with a caliper. A different way of measuring is by expressing GFP or luciferase in the B16 cells and following their volume with an imaging device such as MRI.

Despite the differences between mouse and human metabolism, possible biomarker candidates found in mice urine and feces may serve as a guide to examine similar compounds in human urine. If the possible biomarker candidates were formed in biochemical processes related to the activity of cancer cells, we can expect to find some overlap between biomarkers found in human and mice.

## 4. Methods

Urine and feces samples from nine mice were collected, twice before (healthy group, *n* = 18) and four times after (cancer group, *n* = 35, one mouse died before the last sample was obtained) subcutaneous injection of B16 melanoma cells. The samples were frozen at −20°C until use. Compounds from these samples were separated and identified by using headspace solid phase microextraction (HS-SPME) and GC/MS. In addition, VOCs above a culture of B16 melanoma cells were also collected and analyzed.

Comparison between VOCs content of the two first samples of each mouse, collected before B16 melanoma cell injection, showed high repetition of peaks in the chromatograms of each mouse. The average amount of repetition between these two measurements was 78 ± 6%. Comparison of peak repetition in the chromatograms of different mouse in the two preinjection samples shows a markedly reduced degree of repetition (see Figure S2 in the Supplementary Material). These findings clearly indicate that the metabolism characteristics of each mouse have large influence on the observed chromatograms. Consequently, we did not use a control group but compared the chromatograms of each mouse after B16 melanoma cell injection to those obtained before injection. Thus, each mouse was used as its own control.

### 4.1. Mice

Female C57BL/6J inbred mice, 12 weeks old and weighing 18–20 g, were obtained from Harlan Laboratories (Jerusalem, Israel). All mice were maintained at the animal resource center of Ben-Gurion University of the Negev, in a controlled environment (illumination, temperature, and humidity) free of specific pathogens. All animal experiments described in this work were approved by the Ben-Gurion University Committee for the Ethical Care and Use of Animals in Experiments. Each individual mouse was kept in a separate cage (9 cages total) under the same housekeeping conditions. The first samples were taken after a week of acclimation (at the age of 13 weeks).

### 4.2. Cells

B16F10 cells were cultured in an RPMI 1640 medium supplemented with 10% heat-inactivated fetal calf serum (FCS), 100 U/mL penicillin, 100 mg/mL streptomycin, and 2 mM L-glutamine (Biological Industries, Israel).

### 4.3. Supernatant Collection

B16F10 cells (10^6^ cells) were seeded in a 10 cm diameter tissue culture dish. After 48 h, the medium was collected and centrifuged to eliminate cells and cell debris, and the cells were harvested by 0.23% trypsin and washed once in medium and two more times in phosphate buffered saline (PBS). The cell pellet was subjected to VOC analysis.

### 4.4. Tumor Challenge

B16F10 cells were harvested by 0.23% trypsin and washed once in medium and two more times in PBS. One hundred microliters, containing a total of 2 × 10^5^ B16F10 cells in PBS, was injected subcutaneously to the left flank of each of nine C57BL/6 mice. Tumor cell growth was monitored by a visual inspection and palpation. Mice with tumors that reached the size of 1.5 cm diameter were sacrificed by CO_2_.

### 4.5. Urine and Feces Collection

**Figure 5:**
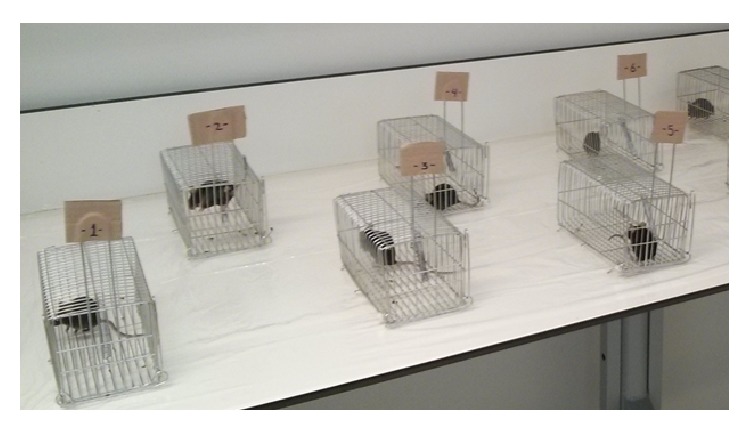
The experimental setup for collecting urine and feces samples.

A new disposable plastic table cover was prepared on each sampling day. The mice were placed in individual cages on the table. Urine and feces samples were collected into vials with a pipette from the new disposable plastic table cover. At the end of the sample collection the cages were cleaned with isopropanol. The experimental system is shown in Figure 5.

We could not find in the literature a component in the urine or feces that allow the prediction of the hydration state of mouse (similar to creatinine in human urine). We found 8 VOCs that showed up in all the chromatograms; however, their peak areas did not show any correlation to the total chromatogram area and they could not be used to monitor hydration state of the mice. Thus, it was assumed that there should not be significant variations in mice hydration state since they were kept in identical conditions (temperature and humidity) with unlimited access to food and water. Moreover, urine and feces samples were collected from all mice at the same time. It should be noted that the amount of urine and feces that were collected from the different mice varied, not enabling a direct comparison between amounts of different VOCs of different mouse.

### 4.6. Contaminants Collection

One vial was left open throughout the experiment to sample room air contaminants. Samples from mouse food, table cover, and sawdust were also collected.

### 4.7. HS-SPME

We used the HS-SPME method to collect volatiles. Static headspace sample extraction was achieved by exposing a 65-*μ*m polydimethylsiloxane/divinylbenzene (PDMS/DVB) SPME fiber (SUPELCO, Bellefonte, PA, USA) to the headspace for 10 min at 60°C. Following the extraction, the fiber assembly was transferred to the GC injection port for desorption at 250°C for 5 min, with the split valve closed for 2 min.

### 4.8. Gas Chromatography-Mass Spectrometry

GC-MS analyses were performed by using an Agilent 6890 series GC system (Agilent, USA) connected to an Agilent 5973 network mass selective detector (Agilent, USA). The bench top system was fitted with an SPME injection sleeve 0.75 mm ID quartz liner (SUPELCO, Bellefonte, PA, USA). The analytical column was a Zebron ZB-5MSi fused-silica capillary column, 30 m × 0.25 mm ID × 0.25 *μ*m film thickness (Phenomenex, Torrance, CA, USA). The carrier gas was 99.9995% pure helium (Maxima, Ashdod, Israel) passed through a moisture trap (model MT-200-2S) and an Agilent hydrocarbon/moisture trap (model HMT200-2) (Agilent, China) at flow rate of 1.0 mL × min^−1^. The GC was operated under the following temperature program: 50°C for 3 min, ramp of 12°C min^−1^ to 240°C, held at 240°C for 5 min, ramp of 50°C min^−1^ to 260°C, then held at 260°C for 1 min, giving a total run of 25.23 min. For GC-MS, following electron ionization, ions were scanned as the total ion current (range: 10–500* m/z* at 2.97 scans × s^−1^). At both the beginning and end of each GC/MS analysis session the headspace over a calibration mixture (containing equal amounts of chloroform, toluene, and dodecanethiol) was used to ensure that the system operation did not change. The test is based on examination of retention time and peak shape of the solvents used in the calibration mixture.

Compounds were assigned a chemical identification by means of spectral library matching, using the NIST'08 and Wiley libraries. The databases search was performed using the ChemStation Software (by Agilent) that was used to control and operate the GC/MS system. The assignment of chemical identity by the software is accompanied by a quality factor (QF). The QF is also termed in some cases as matching factor. A library search procedure that identifies structural features of an unknown compound is carried out. This procedure first retrieves library compounds whose spectra are most similar to the spectrum of the unknown compound. The algorithm then deduces structural features of the unknown compound from the chemical structures of the retrievals. The significance and reliability of each retrieved spectrum are weighted according to its similarity to the spectrum of the unknown compound. There are different procedures to estimate the reliability of the chemical identification of a GC peak (see, e.g., Stein [17]). The exact algorithm used to obtain the QF in the ChemStation Software is not described in the documentation (it is considered as a commercial secret); however, the manual states that QF larger than 80% is considered to be reliable. We accepted assignments as reliable only if their QF was greater than 80%. Below this value of QF the reliability of the assignment is too low and the peaks are designated by their retention time only.

### 4.9. Chromatogram Preprocessing

A GC-MS analysis produces a complex chromatogram, where each peak represents a different volatile compound and the area below the peak is proportional to the amount of the compound in the sample. Peaks area calculations were performed by the software using a predefined threshold value. This threshold was used to ensure that all volatiles with low concentration are detected but “noise” is eliminated. To obtain estimates of the concentration of each compound, the peak area is divided by the total area of all the peaks in the chromatogram. This normalization is performed by the ChemStation Software (by Agilent) used to operate the GC/MS. The total chromatogram area used in the normalization is available in the output file; hence, the actual peak area of any individual feature can be calculated. Examination of the variations in total chromatogram area shows that the standard deviation is 17% of the mean value, a quite narrow distribution. The data analysis described in the present study is based on the normalized peak area values. We repeated some of the analysis using the actual area of the peaks in the chromatograms and found almost identical results to those obtained by the normalized data.

Some of the volatiles in the chromatogram were eluted at multiple RTs and, therefore, we used only the RT with the best match. In addition, to overcome peak shifting across different chromatograms, we first grouped peaks in the same time window (±0.3 s) and then grouped adjacent peaks in adjacent time windows that hit the same match. Each identified volatile was compared to all the background volatiles (room air, plastic table cover, vial, vial septum, SPME fiber, sawdust, and mouse food) to ensure that differences were not due to a background alternation.

### 4.10. Statistical Analysis

A partial least squares discriminant analysis (PLS-DA) was conducted with the SIMCA P+ code (Version 12.0.1.0, by Umetrics AB, Umeå, Sweden) to test whether mice with melanoma could be diagnosed by using the potential urine or feces biomarkers. Each chromatogram vector was defined as a member in one of three classes, “healthy,” “early-melanoma,” and “late melanoma” in urine samples, and in one of two classes, “healthy” and “melanoma” in the feces samples.

### 4.11. MRI

Two mice chosen arbitrarily were scanned three days after the last sample was taken to confirm the existence of the tumor and to estimate its volume. T1-weighted scanning images were acquired at 1T by means of an Aspect M2 high performance MRI system (Aspect Imaging Technologies Ltd., Israel), by using a gadolinium-DTPA contrast agent (Dotarem, 1 mL/kg body weight plus 0.05 mL for catheter).

## Supplementary Material

Table S1. Library match of all the potential biomarker candidates. Some of these potential biomarkers have been reported previously by other research groups as being predictive for melanoma and other types of cancer.

## Figures and Tables

**Table 1:** Classification of samples.

Day^1^	Sample identifier	Palpable tumor	Suggested stage^2^
−3	A	no	Healthy
0	B	no	Healthy
4	C	no	Healthy
7	D	no	Early-melanoma
12	E	yes	Late melanoma
13	F	yes	Late melanoma

^1^Relative to the injection of B16 melanoma cells, which was performed immediately after obtaining sample B.

^2^Based on the signature of the VOCs and on tumor palpability.

**Table 2:** VOCs detected in the urine of tumor-bearing mice but not in healthy mice.

Number	RT (min)	Occurrence		
		Mice	Samples^1^	Stages
1	14.54	8/9	11/17	Late
2	18.41	9/9	19/26	Early, late
3	21.70	9/9	14/17	Late

^1^The number of samples in which the compound was detected, as a fraction of the total samples tested at the relevant stages.

**Table 3:** VOCs detected only in the urine of healthy and early-melanoma mice but not in tumor-bearing mice.

Number	RT (min)	Occurrence		
		Mice	Samples^1^	Stages
1	3.72	8/9	20/27	Healthy
2	3.78	9/9	34/36	Healthy, early
3	4.91	8/9	20/36	Healthy, early

^1^The number of samples in which the compound was detected, as a fraction of the total samples tested at the relevant stages.

**Table 4:** VOCs with higher concentrations in the urine of tumor-bearing mice than of healthy mice.

Number	RT (min)	Fold (%)	*P* value^1^			
			H-C	H-E	H-L	E-L
1	6.21	199	**0.03**	**<0.01**	**0.01**	**0.01**
2	6.67	170	**<0.01**	**<0.01**	**<0.01**	**<0.01**
3	8.58	212	**<0.01**	**<0.01**	**<0.01**	0.43
4	8.90	265	**0.02**	—^2^	**0.02**	—^2^
5	10.79	200	**0.01**	0.92	**<0.01**	0.26
6	11.23	218	**0.03**	**<0.01**	0.14	**0.03**
7	12.44	173	**<0.01**	**0.03**	**<0.01**	0.24
8	13.39	175	**<0.01**	**<0.01**	0.06	**0.04**
9	14.95	124	0.36	**<0.01**	0.28	**<0.01**
10	15.30	131	**0.04**	0.09	**0.03**	0.43
11	15.51	144	**<0.01**	**0.04**	**<0.01**	0.07
12	16.76	173	**0.03**	**0.05**	0.16	0.13
13	16.95	143	**<0.01**	**<0.01**	**<0.01**	0.17

^1^Calculated with a two-tailed paired *t*-test. *P* values smaller than 0.05 are marked in bold. H: healthy; E: early-melanoma; L: late melanoma; C: cancer; both E and L.

^2^This compound was not detected in mice bearing an early melanoma.

**Table 5:** VOCs detected in the feces of tumor-bearing but not in healthy mice.

Number	RT (min)	Occurrence		
		Mice	Samples^1^	Stages
1	10.61	9/9	15/17	late
2	20.42	9/9	15/17	late

^1^The number of samples in which the compound was detected, as a fraction of the total samples tested at the relevant stages.

**Table 6:** VOCs with higher or lower concentrations in the feces of tumor-bearing mice than of healthy mice.

Number	RT (min)	Fold (%)	*P* value^1^			
			H-C	H-E	H-L	E-L
1	6.71	223	**<0.01**	0.27	**<0.01**	**<0.01**
2	6.87	186	**<0.01**	0.83	**<0.01**	**<0.01**
3	6.94	132	**<0.01**	0.84	**<0.01**	**<0.01**
4	8.24	163	**<0.01**	0.10	**<0.01**	**0.02**
5	8.40	186	**0.01**	0.07	**0.01**	**0.05**
6	8.58	199	**<0.01**	**0.03**	**0.02**	**0.03**
7	8.67	116	0.84	0.20	0.43	**0.03**
8	10.21	204	**<0.01**	**0.02**	**<0.01**	0.21
9	10.79	135	**0.03**	0.51	**0.01**	**0.02**
10	13.39	120	0.18	**<0.01**	0.55	**<0.01**
11	14.95	122	0.52	**<0.01**	0.13	**<0.01**
12	16.29	86	0.90	0.37	0.59	0.05
13	16.76	149	0.28	0.08	0.70	0.09

^1^Calculated by using a two-tailed paired *t*-test. *P* values smaller than 0.05 are marked in bold. H: healthy; E: early melanoma; L: late melanoma; C: cancer; both E and L.

**Table 7:** VOCs that were detected in the headspace above cultures of B16 melanoma cells.

Number	RT (min)	Sample
1	4.67	Medium plus cells
2	7.43	Medium plus cells
3	7.80	Medium plus cells
4	13.92	Cells
5	14.70	Cells
6	15.01	Medium plus cells
7	15.85	Cells
8	16.76	Cells
9	19.66	Cells, medium plus cells
10	20.17	Medium plus cells
11	20.19	Cells
12	23.78	Cells

**Table 8:** 7-fold cross validation for urine (feces) model.

Prediction	Classification		
	Healthy	Early	Late
Healthy	27 (26)	1 (4)	1 (1)
Early	0 (1)	8 (5)	1 (1)
Late	0 (0)	0 (0)	15 (15)
