# Supplementary material for: Biomarkers for Detection and Monitoring of B16 Melanoma in Mouse Urine and Feces
Source: J Biomark. 2015 Feb 23;2015:841245. doi: 10.1155/2015/841245 (PMC4437384; doi:10.1155/2015/841245)

**Supplementary Information**

**Table S1.** Library match.

| **RT (min)** | **Compound** | **CAS** | **QF (%)** | **Reference** |
| --- | --- | --- | --- | --- |
| **3.72** | Heptane | 142-82-5 | 72 | Lung - [Poli et al. (2005)](#_ENREF_15) |
| **3.78** | 2-Hexanone | 591-78-6 | 93 | Lung - Hanai et al. (2012), [Filipiak et al. (2010)](#_ENREF_5) |
| **4.67** | 2,4-Dimethyl-1-heptene | 19549-87-2 | 93 |  |
| **4.91** | 2-Ethyl-1H-Isoindole-1,3(2H)-dithione | 35373-06-9 | 90 |  |
| **6.21** | Dimethyl sulfone | 67-71-0 | 94 | [Melanoma - Abaffy et al. (2011)](file:///C:\Users\avivsever\AppData\Local\Microsoft\Windows\Temporary%20Internet%20Files\Content.MSO\B8AE28F2.xls#RANGE!_ENREF_1) |
| **6.67** | 6-methyl-3-heptanone | 624-42-0 | 97 | [Lung - Hanai et al. (2012)](file:///C:\Users\avivsever\AppData\Local\Microsoft\Windows\Temporary%20Internet%20Files\Content.MSO\B8AE28F2.xls#RANGE!_ENREF_8) |
| **6.71** | 6-methyl-2-heptanone | 928-68-7 | 94 | [Liver - Xue et al. (2008)](file:///C:\Users\avivsever\AppData\Local\Microsoft\Windows\Temporary%20Internet%20Files\Content.MSO\B8AE28F2.xls#RANGE!_ENREF_21) |
| **6.87** | 5-methyl-2-heptanone | 18217-12-4 | 91 |  |
| **6.94** | Benzaldehyde | 100-52-7 | 97 | Melanoma - Kwak et al. (2013), Lung - Hanai et al. (2012), Hanai and Baba (2013) |
| **7.43** | Decane | 124-18-5 | 93 |  |
| **7.8** | 1-(2-trimethylsiloxy-1,1-dideuteriovinyl)-4-trimethylsiloxy-benzene | 126210-55-7 | 83 |  |
| **8.24** | Benzeneacetaldehyde | 122-78-1 | 91 |  |
| **8.4** | 2-Octenal | 2363-89-5 | 80 |  |
| **8.58** | Acetophenone | 98-86-2 | 95 | Lung - Hanai et al. (2012), Breast - Qi et al. (2010) |
| **8.67** | o-Toluidine | 95-53-4 | 97 | [Lung - Phillips et al. (1999)](file:///C:\Users\avivsever\AppData\Local\Microsoft\Windows\Temporary%20Internet%20Files\Content.MSO\B8AE28F2.xls#RANGE!_ENREF_13) |
| **8.9** | 4-Trimethylsilyl-9,9-dimethyl-9-silafluorene | 58263-56-2 | 72 |  |
| **10.21** | 2-Decanone | 693-54-9 | 95 |  |
| **10.61** | 9H-pyrrolo[3'',4'':3,4]pyrrolo[2,1-a]phthalazine-9,11(10H)-dione,10-ethyl-8-phenyl | 95647-39-5 | 78 |  |
| **10.79** | Diethoxymethane | 462-95-3 | 86 |  |
| **11.23** | 1,2,3,4-Tetrahydroquinoline | 635-46-1 | 94 |  |
| **12.44** | Propanoic acid, 2-methyl-, 3-hydroxy-2,4,4-trimethylpentyl ester | 74367-34-3 | 90 |  |
| **13.39** | 2,6-di(t-butyl)-4-hydroxy-4-methyl-2,5-cyclohexadien-1-one | 10396-80-2 | 98 |  |
| **13.92** | 2,4-bis(1,1-dimethylethyl)-phenol | 96-76-4 | 94 |  |
| **14.54** | 1-Hexadecanol | 36653-82-4 | 93 | Melanoma - [Abaffy et al. (2011)](#_ENREF_1), Colorectal - [Chan et al. (2009)](#_ENREF_3) |
| **14.7** | Hexadecane | 544-76-3 | 95 |  |
| **14.95** | Isopropyl dodecanoate | 10233-13-3 | 99 |  |
| **15.01** | 7,10-Epoxy-7H-cyclohepta[d]tetrazolo[1,5-b]pyridazine, 6-chloro-10,11-dihydro-11,11-dimethyl | 112998-27-3 | 80 |  |
| **15.3** | Methyl dihydrojasmnate | 24851-98-7 | 98 |  |
| **15.51** | Cyclotetradecane | 295-17-0 | 99 |  |
| **15.85** | Hexadecanal | 629-80-1 | 87 |  |
| **16.29** | Cyclododecene | 1501-82-2 | 95 |  |
| **16.76** | Isopropyl myristate | 110-27-0 | 99 | [Breast - Phillips et al. (2006)](file:///C:\Users\avivsever\AppData\Local\Microsoft\Windows\Temporary%20Internet%20Files\Content.MSO\B8AE28F2.xls#RANGE!_ENREF_12) |
| **16.95** | 6,10,14-Trimethyl-2-pentadecanone | 502-69-2 | 99 |  |
| **18.41** | Isopropyl palmitate | 142-91-6 | 72 | Melanoma - [Abaffy et al. (2011)](#_ENREF_1) |
| **19.66** | 4-(3,4-Dimethoxybenzylidene)-1-(4-nitrophenyl)-3-phenyl-2-pyrazolin-5-one | Unknown | 95 |  |
| **20.17** | 4,4''-(1-methylethylidene)bis-phenol | 80-05-7 | 86 |  |
| **20.19** | Merochlorophaeic acid | 2879-80-3 | 80 |  |
| **20.42** | Ethyl 4-(4-methylphenyl)-4-penteanoate | 32623-17-9 | 60 |  |
| **21.7** | Dehydroabietic acid | 1740-19-8 | 81 |  |
| **23.78** | 2-Oxo-4-nitrosomethyl-6-trifluoro-methyl-1,2-dihydropyrimidine | Unknown | 90 |  |

**Figure S1.** 3D T1-weighted MRI scanning of two mice, 16 days after melanoma cells injection. Lesions are marked in red. Lesions size are approximately 1580 mm^3^ (left), 1747 mm^3^ (right).


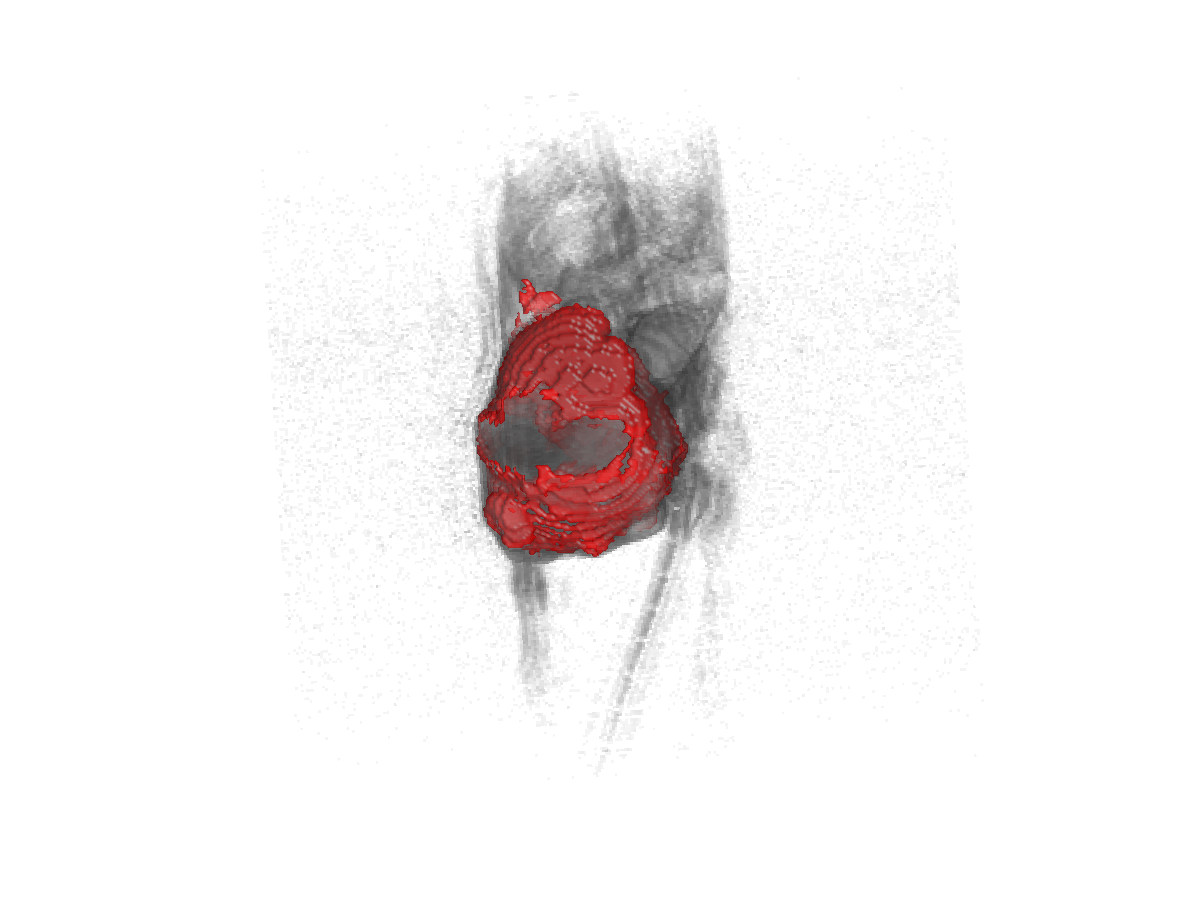

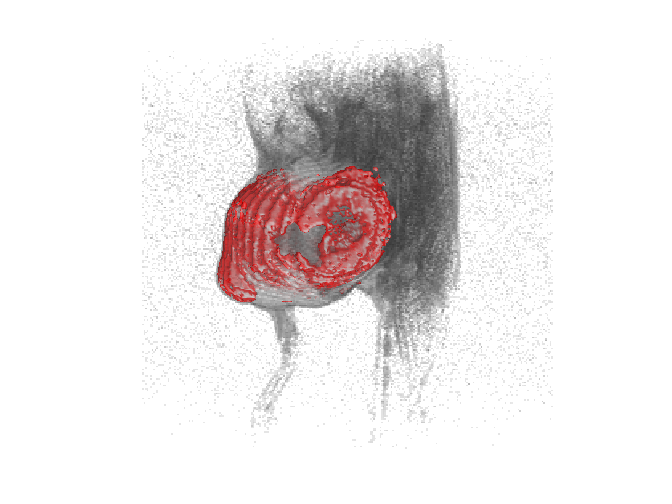


**Figure S2:** Comparison of peak repeatability among the 9 mice used in the study. Each one of the detected peaks (bottom line) is marked by a diamond symbol at the level of its repeatability.


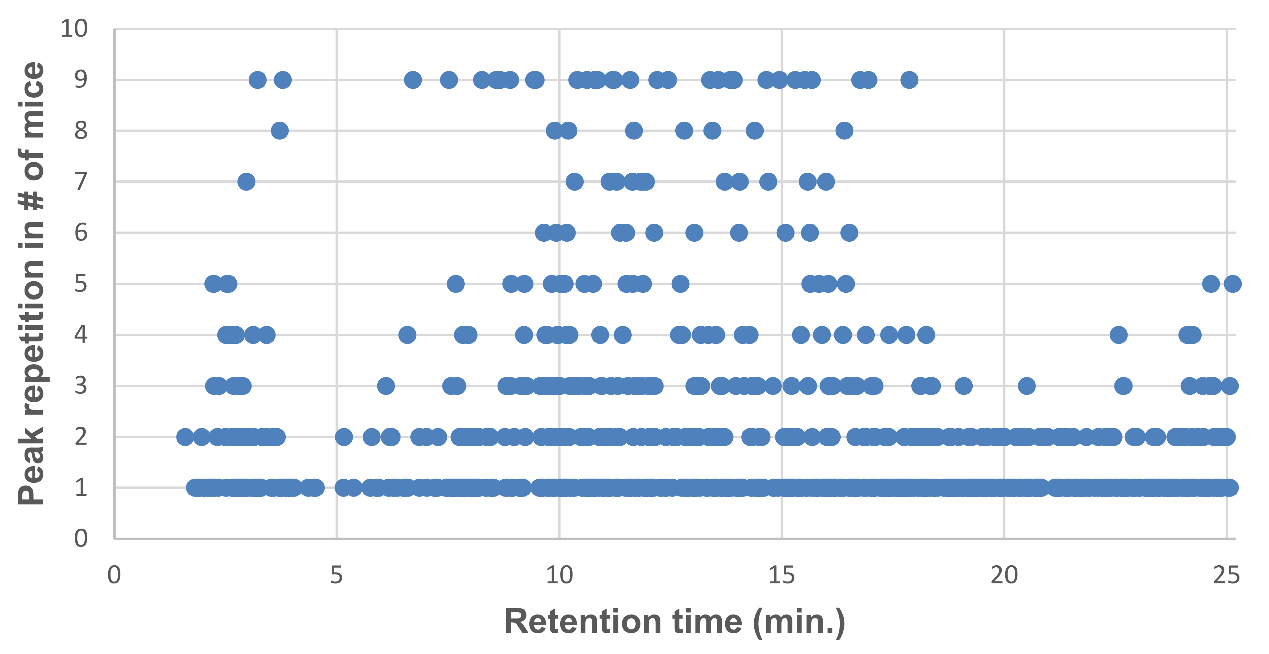

Supplement: Supplementary file 1 — Table S1. Library match of all the potential biomarker candidates. Some of these potential biomarkers have been reported previously by other research groups as being predictive for melanoma and other types of cancer. [file 841245.f1.docx]
